# Development and psychometric evaluation of the sexual satisfaction questionnaire for postmenopausal women (PWSSQ): an exploratory mixed method study protocol

**DOI:** 10.1186/s12978-024-01840-y

**Published:** 2024-07-03

**Authors:** Nasim Shahrahmani, Raheleh Babazadeh, Abbas Ebadi

**Affiliations:** 1 Department of Midwifery and Reproductive Health, Student Research Committee, School of Nursing and Midwifery, Mashhad University of Medical Sciences, Mashhad, Iran; 2 Nursing and Midwifery Care Research Center, Mashhad University of Medical Sciences, Mashhad, Iran; 3 Department of Midwifery, School of Nursing and Midwifery, Mashhad University of Medical Sciences, Mashhad, Iran; 4 Behavioral Sciences Research Center, Lifestyle Institute, Baqiyatallah University of Medical Sciences, Tehran, Iran; 5 Nursing Faculty, Baqiyatallah University of Medical Sciences, Tehran, Iran

**Keywords:** Study protocol, Validity, Reliability, Sequential exploratory mixed-method study, Psychometric evaluation, Postmenopausal women, Sexual satisfaction

## Abstract

**Background:**

The concept of sexual satisfaction in menopausal women is very different from that in premenopausal women, and this difference is due to aging and physical, hormonal, cultural, and psychological changes. Therefore, the first step in discovering methods for assessing sexual satisfaction in postmenopausal women is to develop a measurement instrument. This study was conducted to develop and evaluate the psychometric properties of a sexual satisfaction instrument for postmenopausal women.

**Methods:**

The current study is an exploratory-sequential mixed-methods research project that will be divided into two parts: qualitative and quantitative. Aligned with the primary objective of the research, which is to elucidate the concept of sexual satisfaction in postmenopausal women, the hybrid concept analysis model developed by Schwartz and Kim will be employed. This model comprises three key phases: the theoretical phase, the fieldwork phase, and the final analytical phase. Those who met the inclusion criteria and exhibited maximum variance in terms of age, educational level, employment status, and menopausal duration were recruited. The conventional content analysis will be carried out following the steps proposed by Graneheim and Lundman. Second, in the quantitative phase, the psychometric properties of the instrument were evaluated, including the content, face and construct validity and reliability via internal consistency and stability. The psychometric properties described in the COSMIN checklist will be utilized for designing the instrument.

**Discussion:**

A valid and reliable scale for evaluating the sexual satisfaction of postmenopausal women should be developed, and educational content should be designed to improve the sexual satisfaction of this group of women.

## Abstract (plain language summary)

Menopause is a natural event that is accompanied by numerous physical and psychological changes that create a complex period in the life of postmenopausal women. Sexual satisfaction is a component related to human sexuality and is known as the last stage of the sexual response cycle. Sexual satisfaction is defined as the emotional response resulting from the mental evaluation of positive and negative things in a sexual relationship. Sexual satisfaction is one of the important factors of satisfaction in married life. People who have sexual satisfaction have a significantly better quality of life than those who do not have sexual satisfaction. The concept of sexual satisfaction in menopausal women is very different from that in premenopausal women, and this difference is due to aging and physical, hormonal, cultural, and psychological changes. Sexual satisfaction is important for researchers for two reasons. First, sexual satisfaction provides a mechanism through which to assess a relationship partner's performance. Second, sexual satisfaction is a predictor of other aspects of the relationship, such as marital quality and stability. To discuss feelings and discover methods for achieving sexual satisfaction in postmenopausal women, it is necessary to understand the factors affecting sexual satisfaction and dissatisfaction in this group. The existing tools in the field of women's sexual satisfaction are not designed for this age group (menopausal women) and do not have the necessary comprehensiveness and adequacy to assess sexual satisfaction in menopausal women. Therefore, this study will be conducted to develop and evaluate the psychometric properties of the sexual satisfaction of postmenopausal women.

## Background

Menopause is a natural biological process that affects the physical, mental, and sexual life of women [1]. The WHO predicts that there will be a menopause explosion in 2030, with approximately 1.2 billion women aged over 50 years. Most of them (approximately 80%) live in developing countries. The population of postmenopausal women is increasing by approximately 3% every year [2].

Although sexual performance generally decreases with increasing age, in the study of Addis et al. (2006), the sexual activities and behaviors of 2109 women aged 40 to 69 years were examined, and it was found that approximately 75% of them were sexually active [3]. In many societies, cultural concepts and experiences affect a woman's perception of menopause and her sexual performance during this period. For some women, menopause causes a decrease in sexual activity, and even sexual performance becomes less enjoyable during this time. However, for others, sexual activity during menopause is associated with a sense of freedom due to the lack of fear of pregnancy during this period [4]. One important aspect that is often ignored or considered taboo in the middle-aged and elderly population is sexual satisfaction [5, 6]. Sexual satisfaction is a component related to human sexuality and is known as the last stage of the sexual response cycle. Sexual satisfaction is defined as an emotional response resulting from the mental evaluation of positive and negative things in a sexual relationship [7]. The concept of sexual satisfaction can be divided into two types: satisfaction from sexual activities and emotional-emotional satisfaction [8]. However, other researchers define sexual satisfaction based on individual expectations, that is, the degree of sexual activity that meets the individual's expectations [9]. In fact, sexual satisfaction is considered not only one of the dimensions of sexual health but also a sexual right and the result of sexual well-being and overall health [10, 11]. Satisfaction with sexual relationships is one of the important factors of satisfaction with one’s married life. People who are sexually satisfied report significantly better quality of life than those who are not sexually satisfied [12]. One of the factors influencing sexual satisfaction is estrogen, which plays an essential role in sexual desire. Estrogen plays a role in improving the elasticity of pelvic tissues for sexual intercourse. When estrogen is not produced in a sufficient amount before menopause, vaginal dryness may occur. A low level of estrogen is one of the predictors of sexual dysfunction during menopause [13, 14]. Menopause may directly or indirectly affect women's sexual satisfaction. Vasomotor symptoms associated with menopause, physical symptoms, body image changes, and depression and anxiety resulting from menopause are associated with decreased sexual performance and decreased sexual satisfaction and pleasure [15]. A 2013 review study in France revealed that variables and factors such as a) demographic factors as well as physical and mental health status, b) variables related to intimate relationships and sexual response, c) variables related to social support and family relationships, and d) cultural beliefs and values such as religion all play a role in explaining sexual satisfaction. According to the findings of this study, there is a lack of theoretical models to explain sexual satisfaction and the combination of influencing factors on sexual satisfaction [7]. Sexual satisfaction is important for sexual researchers for two reasons. First, sexual satisfaction provides a mechanism through which to examine relationship partner performance. Second, sexual satisfaction is a predictive structure of other aspects of the relationship, such as marital quality and stability [16]. The concept of sexual satisfaction in menopausal women is very different from that in premenopausal women, and this difference is due to aging and physical, hormonal, cultural, and psychological changes. To determine the methods of achieving sexual satisfaction within the capabilities of a menopausal woman, we need to investigate sexual satisfaction in this special group. The importance of examining the concept of sexual satisfaction from the point of view of menopausal women is necessary for sexual education and sexual therapy because the type of education and the content of education change due to changes in menopause are very different from those in pre-menopause [5, 17, 18]. Menopausal women, as a special group, have special assignments that distinguish them from other age groups to some extent and that affect their health. Therefore, the first step in the development and construction of a measurement tool is to describe the nature of the concept. The more clearly the concept is defined, the easier it is to write its measurement questions [4, 19]. On the other hand, the existing tools in the field of women's sexual satisfaction are not specifically designed for this age group (menopausal women) and do not have the necessary comprehensiveness and adequacy to investigate sexual satisfaction in menopausal women [20, 21]. Therefore, the current study aims to define the concept of sexual satisfaction based on the experiences and views of postmenopausal women, and the studies conducted, based on the hybrid explanatory model, and then, based on the presented concept, to design and psychometrically assess the sexual satisfaction of postmenopausal women. Improving sexual satisfaction in this large and growing population.

### Study aims

The objectives of each phase are as follows.

### Special objectives of the qualitative stage based on the hybrid model (phase 1)

- Explaining the concept of sexual satisfaction from the point of view of postmenopausal women based on articles and qualitative research.
- Identifying the characteristics of the concept of sexual satisfaction from the point of view of postmenopausal women based on articles and qualitative research.
- Identifying the antecedents of the concept of sexual satisfaction from the point of view of postmenopausal women based on articles and qualitative research.
- Identifying the consequences of sexual satisfaction from the point of view of postmenopausal women based on articles and qualitative research.

### Special objectives of the quantitative phase (phase 2)

- Evaluation of the content validity (qualitative and quantitative) of PWSSQ.
- Evaluation of the Face Validity (Qualitative and Quantitative) of PWSSQ.
- Evaluation of the construct validity of PWSSQ using exploratory factor analysis.
- Evaluation of the reliability of PWSSQ.
- The psychometric properties described in the COSMIN.

## Materials and methods

### Study design

This was a sequential exploratory mixed-method study with a qualitative‒quantitative sequencing design. The simultaneous use of quantitative and qualitative methods in a single study is called mixed-method research. The paradigm of this research is pragmatism, in which the researcher collects and analyzes data with two qualitative and quantitative approaches and then integrates and discusses them during a program [22–24]. The current research will be conducted in two qualitative and quantitative parts. The qualitative part will use the approach of hybrid conceptual analysis to explain the concept of sexual satisfaction to compile the primary items, and the quantitative part will address the validity and reliability of the designed tool. The different stages of the research will be designed and carried out as follows.

### Phase 1: qualitative part of the study

In line with the first goal, which is to explain the concept of sexual satisfaction in postmenopausal women, the hybrid concept analysis model was proposed by Schwartz-Barcott & Kim (2000). It consists of three phases: the theoretical phase, the fieldwork phase and the analytic phase [25–27].

### A: Theoretical phase

Hybrid concept analysis, our research methodology, utilizes both theoretical literature analysis and empirical observation to define a concept [28].

This stage includes 4 steps: A-1- Selecting a concept, A-2- Searching the literature, A-3- Dealing with meaning and measurement, and A- 4- Choosing a working definition.

- A-1- Selecting a concept: At this stage, the concept of sexual satisfaction in menopausal women was chosen based on the research goal.
- A-2- Searching the literature: Whittemore and Knafl's integrated review was used to conduct the literature search in this study. The five steps of Whittemore and Knafl's method are as follows: the problem identification stage, literature search, data evaluation, data analysis, and presentation [29].
- A-2- Stage 1. Problem identification stage: This method begins with the precise identification of the issue that requires investigation. The variables of interest (i.e., concepts, target population, health care problem) and the suitable sampling frame (i.e., type of empirical studies, inclusion of theoretical literature) subsequently establish the objective [29]. The concept of "sexual satisfaction of postmenopausal women" was taken into account.
- A-2- Stage 2. Literature search: A comprehensive search for an integrative review typically involves using a minimum of two to three strategies to identify the greatest number of eligible primary sources [29, 30]. The investigation was carried out using the following international databases: Google Scholar, CINAHL, Embase, Medline via OVID, PsychINFO, Web of Science, Cochrane, ScienceDirect, ProQuest, and Scopus. The search keywords used were “postmenopausal”, “menopause”, “postmenopausal women”, “aged”, “older adults”, “older women”, “midlife”, “middle-aged”), “sex* satisfy*”, “satisfy* sex*”, “satisfaction with sex”, “sexual satisfaction”, “dissatisfaction”, “concept”, “meaning”, “dimensions”, “predictors”, and “affecting factors”. Keywords were combined using the Boolean operators “AND” and “OR”.
- A-2- Stage 3. Data evaluation: The full texts of all these articles were assessed using the checklists of the Mixed Methods Appraisal Tool (MMAT) 2018, and the MMAT is a critical appraisal tool designed for the appraisal stage of systematic mixed studies reviews, i.e., reviews that include qualitative, quantitative, and mixed methods studies. It permits us to appraise the methodological quality of five categories of studies: qualitative research, randomized controlled trials, nonrandomized studies, quantitative descriptive studies, and mixed methods studies [31].

The PRISMA 2020 Checklist was utilized for the evaluation of the articles. The PRISMA checklist comprises 27 items that pertain to the content of a systematic review and meta-analysis. It encompasses sections such as abstracts, methods, results, discussion, and financial resources [32]. During the final evaluation stage, articles that were directly relevant to the purpose of the concept analysis stage were chosen. The following questions were also used for eligibility assessment: “Do the articles describe and define sexual satisfaction in postmenopausal women?”, “Do these articles address the characteristics of sexual satisfaction in menopausal women?”.

- A-2- Stage 4. Data analysis: In research studies, data analysis necessitates the sorting, coding, categorizing, and summarizing of information derived from primary sources. Its purpose is to arrive at a unified and cohesive conclusion regarding the research problem and to present the data in a comprehensive and unbiased manner. Integrated surveys utilize the same analysis strategies and data analysis methods. The four steps of this method are data reduction, data display, data comparison, and conclusion drawing and verification [29, 33]. Data reduction will be classified into qualitative, quantitative, review, experimental, and semiexperimental studies. The research data are categorized in tables based on the purpose of the study, the populations under study, and the year of implementation. At this stage, the researcher will move to higher levels of abstraction and classes that should be extracted from the data, including definitions, aspects of characteristic formation, antecedents, and consequences. The concept will be examined, data from primary sources will be compared, and codes will be formed. At this stage, to accurately explain the concept of sexual satisfaction in postmenopausal women, a table of antecedents, consequences, and characteristics will be designed.
- A-2- Stage 5 Data presentation: The concepts of sexual satisfaction are presented in the tables.
- A-3- Dealing with meaning and measurement: the factors of sexual satisfaction in postmenopausal women presented in the articles will be compared in terms of similarities and contradictions.
- A- 4- Choosing a working definition: A concept of sexual satisfaction in postmenopausal women will be expressed from the results of the theoretical stage.

### B: Field work phase

After the theoretical stage, the field work will begin. This stage includes the basic steps that exist in qualitative research. Data collection will begin after receiving the code of ethics from Mashhad University of Medical Sciences. This stage will include a contractual qualitative analysis, which will be carried out in 2024 in the health centers of Kerman Province.

### Sampling and selection of participants

All menopausal women who visit Kerman health centers for medical care will be included in the research population.

### Participants and inclusion criteria

Menopausal women between the ages of 40 and 70 who did not have a known mental illness or limiting physical illness, who did not use sex hormones, who could speak Farsi fluently, who were willing to share their experiences regarding the research topic, and who had engaged in sexual activity with their spouses were included in this study.

### Data collection

The participants will be selected via targeted sampling to ensure maximum diversity in terms of age, menopausal age, education, socioeconomic class, and religious beliefs, and the sampling process will continue until data saturation. After obtaining the necessary permits at health centers, the researcher will explain the study to women who are eligible to participate. If the participants agreed, the data were gathered via semi-structured in-depth interviews with open-ended questions. Individual interviews will be conducted in a quiet room at the health center. Only the participants and the researcher will be present in the interview room. The voices of the interviewees will be recorded with their permission. The interviews will last 30–60 min. None of the participants refused to participate in the study. The first questions in the interviews will be as follows:

- What is your opinion on sex during menopause?
- How has menopause affected your sex?
- How has your sexual satisfaction during menopause changed compared with that before menopause?
- In your opinion, what conditions are effective in increasing your sexual satisfaction after menopause?

### Data analysis

The researcher investigated the participants' statements based on their responses to the questions "Can you explain more?" and "Can you give an example?". The data will be analyzed using both conventional content analysis and the Graneheim and Lundman method [34]. First, the interviews will be transcribed and reviewed several times. The units of analysis will then be extracted and, based on the meaning hidden in them, will progress to the abstraction and conceptualization stages before being named with codes. The codes are divided into subclasses, which are then classified based on their similarities and differences. Finally, the main classes were extracted and identified using the most recent concepts in the transcript.

### Determining the scientific validity and reliability of the data

In qualitative research, the concepts of credibility, dependability, confirmability, and transferability are used to ensure trustworthiness [35]. The researchers' long-term involvement with the subject under study, as well as the allocation of adequate time for data collection, will ensure the validity of the data. Considering that the researcher has several years of experience working in health centers, has positive interactions with women, and has professional experience, she will be able to collect accurate and rich data about the research problem. In addition, the researcher will check the validity of the data with the rest of the research team. Peer review will be another method used to guarantee the consistency of findings (with data reviewed by experts in the field of reproductive health). External reviewers who are not part of the research team but are familiar with qualitative research will evaluate the consistency and reliability of the data. Every step of the research process will be documented, and a report will be created to assess the verifiability of the results. Finally, comprehensive, detailed, and sequential explanations will be implemented to enhance the transferability of the data.

### C: The final analytic phase

In the final analysis stage, the antecedents, characteristics and consequences obtained from the theoretical and practical stage will be compared and combined to provide a precise definition of sexual satisfaction in postmenopausal women that is supported by both the literature and the women's point of view [36].

### Phase 2: the quantitative part of the study

This part is a study of instrument design and validation. To design a tool for measuring the sexual satisfaction of postmenopausal women, sexual satisfaction items will be obtained from the first stage of the research with a hybrid model, and then its validity and reliability will be assessed. The above steps are based on Waltz's model, which includes two sub-studies of instrument design and psychometrics [37].

### Sample size

Based on the psychometric items of the tool at different stages after the initial design of the questionnaire, the small sample size will be determined as follows:

- To assess face validity by qualitative and quantitative methods in 10 postmenopausal women.
- Content validity by quantitative and qualitative methods of 15 psychometric experts and reproductive and sexual health experts.
- The validity direction of the structure of 3 to 10 participants will be determined based on each of the items.
- Retest reliability of 30 postmenopausal women [38].

After the initial design of the sexual satisfaction tool for postmenopausal women, its psychometric properties, including its validity (face, content, and construct) and reliability (internal consistency and stability), will be determined [37].

### Face validity

The face validity of this study will be assessed by quantitative and qualitative methods. To qualitatively determine face validity, the researcher interviewed 10 postmenopausal women face to face and asked their opinions about the level of difficulty, relevancy, and ambiguity of each item. The item impact score is calculated by the following formula:

$$\text{Impact score}=\text{frequency }(\text{\%})\times \text{importance}$$

Importance = Participants who checked options 4 and 5. An impact score greater than 1.5 indicates that the item is acceptable and will be chosen for further analysis [39].

### Content validity

The face validity of this study will be assessed by quantitative and qualitative methods. In the qualitative content validity method, the opinions of 15 experts in the fields of qualitative research, psychology, instrument development, reproductive health, and midwifery will be used to assess the proper grammar, correct words and item scoring, and appropriateness. The quantitative validity of the content will be assessed using the content validity ratio (CVR) and content validity index (CVI). To determine the content validity ratio (CVR), experts will be asked to evaluate the significance of each item. The rating for each item will be assessed on a three-point scale.; Essential (E), useful but not essential (U), not essential (N). Then, the votes of the panel members will be subquantified using the formula. In this formula, ne is the number of experts who consider an item necessary, and N is the total number of panel members. The minimum acceptable value of the CVR will be considered to be 0.49 based on the LawShe Table (1973) [40, 41]. CVR = (Ne—N/2)/(N/2).

The CVI is the most widely reported index for quantifying content validity in instrument development. The CVI will be evaluated based on content validity according to Waltz and Bausell (1983). The expert evaluation focused on clarity, relevance, and simplicity and was expressed using a four-point Likert scale (not relevant, somewhat relevant, quite relevant, highly relevant). The minimum acceptable content validity index will be 0.8 according to Waltz and Bausell (1983). The mean scores of the I-CVIs for all items on the scale will be evaluated using the S-CVI through the average scores for the content validity index. S-CVI values exceeding 0.9 indicate excellent content validity.

### Construct validity

In this research, exploratory and confirmatory factor analysis will be used [37, 42].

### Exploratory factor analysis (EFA)

The goals of exploratory factor analysis are instrument development, theory development, and data reduction [43]. The number of samples required for factor analysis varies from 3–10 samples per item. The number of samples required for factor analysis varies from 3–10 samples per item [38]. Menopausal women between the ages of 40 and 70 who did not have a known mental illness or limiting physical illness, who did not use sex hormones, who could speak Farsi fluently, who were willing to share their experiences regarding the research topic, and who had engaged in sexual activity with their spouses were included in this study. Bartlett’s test of sphericity will be used to determine the operability of the data, and the Kaiser‒Meyer‒Olkin (KMO) index will be used to determine the adequacy of the data. The KMO index ranges from 0 to 1. A KMO greater than 0.7 is interpreted as an acceptable and large sample size that is suitable for EFA. Based on the initial results of the factor analysis, orthogonal and oblique rotation methods were used to better present the data [44]. Factors were named according to the common meaning of the items.

### Confirmatory factor analysis

By performing confirmatory factor analysis, the hypothesized model will be compared with the observed model in the real world. Confirmatory factor analysis will be performed in LISREL software [45].

### Reliability

The reliability of PWSSQ will be assessed using internal consistency and stability measures. Internal consistency will be evaluated by calculating the Cronbach’s alpha coefficient for PWSSQ and its subcategories. An alpha value of 0.70 or higher was deemed acceptable. The test–retest reliability of PWSSQ and its subcategories over a two-week period will be determined using the intraclass correlation coefficient (ICC), with ICCs between 0.7 and 0.8 indicating adequate stability. Items that demonstrate poor reliability will be excluded from factor analysis to ensure construct validity. The instrument will be designed based on the psychometric properties outlined in the Consensus-based Standards for the Selection of Health Status Measurement Instruments (COSMIN) checklist.

## Discussion

Menopause refers to the permanent cessation of menstruation. Women in developed countries live approximately 30 years, or more than one-third, of their lives during the postmenopausal period [46]. Sexual intercourse is an essential component of women's health care at all ages because sexual dissatisfaction and general well-being are closely related. Estrogen and androgen deprivation may manifest with multisystem consequences, including effects on tissues devoted to sexual function [47]. Vaginal dryness, bleeding during intercourse, and dyspareunia are all common complaints of menopausal women, and these symptoms on average lead to decreased libido and decreased sexual satisfaction in menopausal women [48, 49]. Understanding the concept of different aspects of sexual concepts is necessary for the correct assessment of women's health, but due to the multiple nature of sexual reality, scientific truths cannot be obtained without discovering its dimensions in a natural context [11, 50, 51]. Therefore, the concept of sexual satisfaction in postmenopausal women is very different from that in premenopausal women [17].

To discover the methods of achieving sexual satisfaction within the capabilities of a menopausal woman, we need to investigate sexual satisfaction in this special group because the type of training and the content of training are very different due to the changes in menopause compared to pre-menopause. In fact, evaluating the interaction between various predisposing, accelerating, and maintaining sexual satisfaction factors requires extensive analysis. When a single questionnaire is used for these different age groups, the questions asked may not accurately measure the concept of sexual satisfaction in postmenopausal women because the definition of the concept of sexual satisfaction in postmenopausal women is different from that in premenopausal women.

In recent decades, research on women's sexual satisfaction has attracted much attention, and various tools have been used to measure it. One of the most widely used tools in the world and Iran is the sexual satisfaction tool of Larson et al. (1998) [52]. This tool is not specific for women during menopause, and this tool is old. In addition to the fact that some items overlap with sexual performance and do not distinguish between sexual satisfaction and distress, it is suitable for assessing romantic relationships. Additionally, other tools were not designed for menopausal women and are outdated, sexual expression changes over time, and information needs are necessary according to these changes [53]. It is expected that by designing this tool and evaluating the sexual satisfaction of postmenopausal women, it will be possible to help the planners and providers of health services and specialists of different scientific groups with proper planning, appropriate allocation of resources and facilities based on the reported priorities to improve the sexual satisfaction of postmenopausal women and, ultimately, help improve the sexual health of women during menopause, the number of whom is increasing daily. The strength of this study is the use of the hybrid conceptual method in the first stage of the study, which will provide comprehensive information for the design of the desired tool items. This protocol has certain limitations similar to those of other qualitative approaches, such as sampling only in Iran and limited generalizability, which can be reduced with maximum diversity in sampling. They should not talk about their private sexual issues, which should be done by assuring them of confidentiality to reduce this challenge.

## Data Availability

No datasets were generated or analyzed during the current study.

## Abbreviations

PWSSQ: Postmenopausal Women's Sexual Satisfaction Questionnaire
WHO: World Health Organization
CVR: Content Validity Ratio
CVI: Content Validity Index
EFA: Exploratory Factor Analysis
KMO: Kaiser–Meyer–Olkin
ICC: Intraclass Correlation Coefficient
COSMIN: Consensus-based Standards for the Selection of Health Measurement Instruments
